# Applying value-based strategies to accelerate access to novel cancer medications: guidance from the Oncology Health Economics Expert Panel in Qatar (Q-OHEP)

**DOI:** 10.1186/s12913-022-08981-5

**Published:** 2023-01-06

**Authors:** Anas Hamad, Shereen Elazzazy, Salha Bujassoum, Kakil Rasul, Javid Gaziev, Honar Cherif, Zakiya Al-Boloshi, Yolande Hanssens, Ayman Saleh, Hadi Abu Rasheed, Daoud Al-Badriyeh, Ahmed Babiker, Amid Abu Hmaidan, Moza Al-Hail

**Affiliations:** 1 Pharmacy Department, National Center for Cancer Care & Research, Hamad Medical Corporation, PO Box 3050, Doha, Qatar; 2 Medical Oncology Department, National Center for Cancer Care & Research, Hamad Medical Corporation, PO Box 3050, Doha, Qatar; 3 Hematology Department, National Center for Cancer Care & Research, Hamad Medical Corporation, PO Box 3050, Doha, Qatar; 4 Drug Supply Department, Hamad Medical Corporation, PO Box 3050, Doha, Qatar; 5 Pharmacy Executive Office, Hamad Medical Corporation, PO Box 3050, Doha, Qatar; 6 Division of Pediatric Hematology/Oncology, Sidra Medicine, PO Box 26999, Doha, Qatar; 7 Professional Development & Scientific Research Department, Qatar Cancer Society, PO Box 22944, Doha, Qatar; 8 College of Pharmacy, QU Health, Qatar University, PO Box 2713, Doha, Qatar; 9 Registration & Drugs Pricing Section, Pharmacy & Drug Control Department, Ministry of Public Health, PO Box 42, Doha, Qatar; 10 National Cancer Program, Directorate of Policy, Ministry of Public Health, PO Box 42, Doha, Qatar

**Keywords:** Patient access, Patient-reported outcomes, Cancer, Hematology, Oncology, Precision medicine, Health economics, Pharmaceutical procurement, Health policy, Qatar

## Abstract

**Background:**

In line with global trends, cancer incidence and mortality may have decreased for specific types of cancer in Qatar. However, the cancer-related burden on patients, healthcare systems, and the economy is expected to expand; thus, cancer remains a significant public healthcare issue in Qatar. Qatar’s free access to cancer care represents a considerable economic burden. Ensuring the best utilization of financial resources in the healthcare sector is important to provide unified and fair access to cancer care for all patients. Experts from the Qatar Oncology Health Economics Expert Panel (Q-OHEP) aimed to establish a consistent and robust base for evaluating oncology/hematology medications; involve patients’ insights to accelerate access to cutting-edge medications; increase the value of cancer care; and reach a consensus for using cost-effective strategies and efficient methodologies in cancer treatment.

**Methods:**

The Q-OHEP convened on 30 November 2021 for a 3-hour meeting to discuss cancer management, therapeutics, and health economics in Qatar, focusing on four domains: (1) regulatory, (2) procurement, (3) treatment, and (4) patients. Discussions, guided by a moderator, focused on a list of suggested open-ended questions.

**Results:**

Some of the salient recommendations included the development of a formal, fast-track, preliminary approval pathway for drugs needed by patients with severe disease or in critical condition; and encouraging and promoting the conduct of local clinical trials and real-world observational studies using existing registry data. The Q-OHEP also recommended implementing a forecast system using treatment center data based on the supply/demand of formulary oncology drugs to detect treatment patterns, estimate needs, expedite procurement, and prevent shortages/delays. Furthermore, the panel discussed the needs to define value concerning cancer treatment in Qatar, implement value-based models for reimbursement decision-making such as health technology assessment and multiple-criteria decision analysis, and promote patient education and involvement/feedback in developing and implementing cancer management guidelines.

**Conclusion:**

Herein, we summarize the first Q-OHEP consensus recommendations, which aim to provide a solid basis for evaluating, registering, and approving new cancer medications to accelerate patient access to novel cancer treatments in Qatar; promote/facilitate the adoption and collection of patient-reported outcomes; and implement value-based cancer care in Qatar.

## Background

Cancer incidence and mortality differ by cancer type, sex, ethnicity, country, and residence in rural or urban areas. Although some high-income countries and regions, such as the United States and Europe, have reported lower cancer mortality in recent decades, for some specific types, such as lung cancer [1], cancer is still the leading cause of death in countries of high and middle income. Furthermore, as the incidence of some cancers continues to increase globally, translating into an increased burden of cancer-related morbidity [2], the burden of cancer is expected to continue to expand in countries of all income levels [3]. By 2040, the global cancer burden is expected to reach 28.4 million cases, representing an increase of 47% from 2020; this is largely attributable to a rise in risk factors associated with economic development and globalization [2].

In Qatar, the incidence of cancer seems to increase with age, and between 2002 and 2006, the total number of cancer cases was reported to have increased by 57% from the period of 1991 to 1996 [4]. This increase can be attributed in part to earlier diagnosis in recent years, as Qatar introduced national screening programs for breast and colorectal cancers in 2016, as well as the rapid expansion of Qatar’s population [5]. However, the incidence per 100,000 population was considerably lower in Qatar than in other Middle Eastern countries [4]. The Qatar National Cancer Registry at the Ministry of Public Health reported 2,137 new cancer cases diagnosed during 2018, out of a population of nearly 2.7 million. The most reported cancers were breast, colorectal, prostate, leukemia, and non-Hodgkin lymphoma [6]. As with global trends, it is suspected that mortality for specific types of cancer is also decreasing in Qatar [7–9]. Nevertheless, the related burden on patients, healthcare systems, and the economy illustrates that cancer is a significant public health issue in Qatar [4].

Qatar provides cancer care to all citizens and residents free of charge. Such care includes acute and ambulatory care, laboratory tests, clinical imaging, systemic anti-cancer therapies, surgery, and radiotherapy. This free access results in considerable spending on cancer care, which requires strong governance to avoid waste, enhance efficiency, and provide value-based care. Although health insurance will be mandatory for all Qatar residents and visitors by 2023, it is necessary to ensure the best utilization of financial resources in the healthcare sector. This is particularly important to ensure that patients with cancer have unified and fair access to the most effective and novel medical therapies.

Many novel drugs have been developed in recent years, but many of these tend to be costly despite unresolved questions of safety and efficacy. Several cancer medicines are approved based on early-phase short-term clinical trials with small patient samples that demonstrate improvement of surrogate markers, rather than tangible clinical outcomes such as progression-free survival and overall survival [10]. Thus, there may be ethical issues in using such drugs to treat cancer patients with respect to the risk–benefit balance. Nevertheless, preliminary approval pathways may benefit patients with difficult-to-treat, refractory, or resistant cancers, for which treatment options are limited. It is essential to have the appropriate tools available at all cancer treatment centers to ensure patients undergo appropriate biomarker testing to optimize treatment selection and apply precision medicine, which leads to improved treatment outcomes and possibly lower treatment costs [11].

Value-based care is a novel concept in oncology management [12]. Value of care has been defined as the health outcome achieved per dollar spent [13]. Such health outcomes are determined by the healthcare provider and the patient, namely, patient-reported outcomes [12]. By assessing these outcomes, a higher value of care may be achieved for patients, which should ultimately be the goal of healthcare systems [13]. The National Cancer Framework (NCF) 2017–2022 for cancer care in Qatar [14], based on the National Cancer Strategy 2011–2016 (NCS), aims to improve the quality and delivery of cancer care by improving the understanding of patients’ needs. The NCF includes nine specific domains, provides guidance on recommended activities, and specifies success measures to develop implementation programs and measure the program outcomes. The Qatar Oncology Health Economics Expert Panel (Q-OHEP) is a collaborative project that involved interdisciplinary presentation of different specialties including physicians (oncologists and hematologists), pharmacists (practitioners and payers), academicians, and administrators. Authors were selected as seniors in their fields with representation from the main healthcare facilities and organizations in Qatar including Hamad Medical Corporation (HMC) Corporate Pharmacy, National Center for Cancer Care and Research (NCCCR), Qatar University (QU), Ministry of Public Health (MOPH), Qatar Cancer Society (QCS), and Sidra Medicine. The main objectives of the panel were to establish a consistent and robust base for evaluating oncology/hematology medications; involve patients’ insights (i.e., patient-reported outcomes) to accelerate access to cutting-edge medications; and increase the value of cancer care in Qatar. Further, the panel aimed to build a structured guidance/reach a consensus for using cost-effective strategies and efficient methodologies in cancer treatment. The present paper summarizes the methodology used and the key consensus-based recommendations of the Q-OHEP.

## Methods

The Q-OHEP, comprising members of the HMC, NCCCR, MOPH, QU, QCS, and Sidra Medicine, convened on 30 November 2021 for a 3-hour meeting to discuss several aspects of cancer management, therapeutics, and health economics in Qatar, following the objectives listed above. Discussions focused on four domains based on previously published literature on similar experiences [15] as well as adaptations from guidelines for the formulation of consensus-based recommendations [16]: (1) regulatory, (2) procurement, (3) treatment, and (4) patients. Discussions, guided by a moderator, were focused on a list of suggested open-ended questions (Table 1). Ethical approval was not required for this study, as confirmed by HMC’s Institutional Review Board, because it did not include any patient data or extract any information from specific datasets. All participating experts accepted to have the discussion voice recorded.

**Table 1:** Discussion questions for the Qatar Oncology Health Economics Expert Panel (Q-OHEP) roundtable discussion, held on 30 November 2021

Domain	Questions
Regulatory	• What are the oncology regulatory challenges with the current approval process? • How can we accelerate effective registration and use of oncology/hematology medications in Qatar? • Is a preliminary approval pathway in place? • Regarding clinical trials, what are the main challenges of these processes/protocols?
Procurement	• What procurement issues do you or your institution experience in terms of accessibility to oncology medications? • What are the current challenges to fair access to oncology/hematology medications? • What are the available value-based options in Qatar, and what other models might be adopted?
Treatment	• What are the challenges in applying/adopting national/international guidelines? • What are the key barriers you see for the use of new treatments in Qatar? • What are the current major treatment burdens for patients?
Patients	• What are the systemic barriers to patient involvement in medication management or clinical trials? • How can we raise awareness among patients about involvement or participation in clinical trials? • Can you share a story that highlights an aspect relevant to accelerating access to medications?

## Results

### Regulatory Domain

Registration of novel cancer drugs in Qatar is fast-tracked via the MOPH Pharmacy and Drug Control Department, making them available as registered products in the State of Qatar as part of the Qatar National Formulary (QNF). HMC, as the primary provider of oncology care in Qatar, along with Sidra Medicine, prescribes the majority of oncology medications and has the autonomy, in certain conditions, to use drugs even if they are not registered yet by the MOPH. However, preapproval is required from the local pharmacy and therapeutics (P&T) committee at NCCCR, followed by the HMC corporate P&T committee. This approval is usually expedited, making these drugs available in a timely fashion to be prescribed by authorized prescribers in the country.

HMC can purchase unregistered medications if necessary. This can be beneficial in oncology as the new drug registration process by the Pharmacy and Drug Control Department can be protracted due to limited data for a quick decision and the complexity of pricing. However, in many cases, patients needing such medications require them urgently. Drugs that are not available in the HMC formulary are handled as non-formulary items. These drugs should already be approved by the United States Food and Drug Administration (FDA) or European Medicines Agency (EMA). These drugs should be included in specialized society guidelines (e.g., the National Comprehensive Cancer Network [NCCN]) and thus be considered established therapy. In a few exceptional cases, the compassionate use of unregistered drugs in Qatar may be considered. When compassionate use is granted, the cost is covered by the manufacturer.

#### Regulatory challenges with the current approval process

There are several regulatory challenges related to Qatar’s current drug approval process. Although the time required for these processes varies by therapeutic area, drug registration and approval processes are generally long (from 6 to 12 months). However, some drugs can be lifesaving even in a few cases, so it is necessary to expedite their purchase. Therefore, cancer products are granted high priority for approval as formulary items in Qatar by HMC based on the unmet needs of patients.

Another challenge is the uncertainty of the efficacy and safety of new drugs, especially those with FDA accelerated approvals, making it difficult for regulatory agencies to evaluate these drugs based on limited data. Pricing is another important challenge, as it is difficult to determine the most appropriate price, particularly for drugs with multiple indications or those that need to be administered in combination with other drugs. In addition, reference pricing is not always feasible as there may be a lack of transparency in terms of pricing across the Gulf Cooperation Council region, and some neighboring countries with larger populations may get better prices.

Key recommendations:

1. Promptly collect and provide more robust clinical data to shorten the regulatory approval process.
2. Continue to test and gain experience with the drug as a non-formulary item to then provide first-hand clinical evidence to both the local and corporate P&T committees, which may lead to faster approval.
3. Provide incentives for quick registration such as the exclusivity of the market, competitive pricing, or preferential treatment.
4. Involve the Qatar Cancer Research Partnership, a government-established committee that regularly conducts evaluations of the challenges, risks, and other cancer-related issues from the patient perspective.

#### Acceleration of an effective approval process

Oncology-specific P&T committee at NCCCR, along with Sidra Medicine, provide the management of cancer in Qatar and handle the evaluations and recommendations related to the inclusion of drugs in the HMC formulary, which is supported by a fast-track/priority process at the corporate P&T level to accelerate the effective approval (and hence, use) of oncology medications in Qatar.

Key recommendations:

1. Give fast track/priority to at least one drug of each class as a formulary item, particularly drugs that have been approved by the FDA or EMA and are recommended in international treatment guidelines.

#### Preliminary approval pathways

Although oncology products are granted high priority for approval as formulary items in Qatar, there is currently no preliminary drug approval pathway in place. Of note, any oncology product submitted for registration to the MOPH will be given fast-track assessment and approval even if HMC does not request it. However, the product needs to be registered in the country of origin and used according to clinical evidence.

Key recommendations:

1. Develop a formal, fast-track, preliminary approval pathway, especially for drugs needed for cancer patients with severe disease or in critical condition.

#### Main challenges in clinical trials

The main challenges in terms of clinical trials at the national level include the following: The level of awareness of patients and their families regarding the need and value of being included in clinical trials, especially for cancer medications, has not been assessed. The number of patients for enrollment in clinical trials is small, and there is limited availability of reference laboratories. Furthermore, several patients seek treatment abroad. Finally, enrollment of patients from Qatar in international randomized clinical trials is very limited.

Key recommendations:

1. Measure the level of awareness of cancer patients and their families in Qatar regarding the need and value of clinical trials.
2. Conduct awareness campaigns about the importance of clinical trials among patients with cancer and their families using multi-method approaches, including pamphlets, videos, and support groups.
3. Reduce the time taken for processing applications for initiating clinical trials.
4. Work at the level of healthcare providers to encourage enrollment in clinical trials.
5. Establish partnerships with international groups, such as the European Organization for the Research and Treatment of Cancer or the American Association for Cancer Research, and facilitate the participation of cancer patients in Qatar in international studies or multi-site trials to help mitigate the low sample sizes, as well as other limitations of conducting clinical trials in Qatar.
6. Prepare the infrastructure to support the conduct of clinical trials that do not rely entirely on the treating physician; i.e., training of dedicated research nurses, research pharmacists, and research assistants.
7. Encourage/promote the conduct of more local studies, not only clinical trials but also observational studies based on existing registry data to help clarify the efficacy and toxicity of treatments and other safety issues in Qatar, and so transform the available real-world data into local real-world evidence.
8. Conduct pharmacogenomic studies using Qatar Genome Programme and Qatar Biobank data to identify genetic factors relevant to drug efficacy or toxicity.

### Procurement domain

#### Accessibility to cancer medications

Several procurement issues have been experienced regarding accessibility to cancer medications in Qatar. Most drugs are obtained via group purchasing. Other conventional medications can be obtained through local suppliers or neighboring countries. Non-formulary medications, especially those for rare diseases, are bought from wholesalers abroad with long leading times. In urgent life-saving cases, the medical attachés of Qatar embassies abroad are enlisted to help. Thus, there are challenges related to the complexity of the supply chain model used in the Middle East. Most pharmaceutical companies have no large warehouses, resulting in further delays in procurement, drug supply, and supply chain management. However, during the height of the COVID-19 pandemic, the main challenges faced were extended lead times due to disrupted supply chains and airport closures. Notably, many Qatari individuals who were treated abroad returned to Qatar, and the oncology treatment demand rose, resulting in higher use of drugs.

Blanket purchase agreements (usually valid for 3 years) are in place with most international manufacturers, which maintain stable pricing, and allow the request of drugs on demand rather than holding large stocks. However, there are issues with the types of procurement agreements currently in place with some pharmaceutical companies, mainly financial agreements based on reference pricing. It is necessary to obtain a specific volume of drugs to obtain more accessible pricing.

Key recommendations:

1. Implement a forecast system using data collected from treatment centers based on the supply and demand of oncology drugs listed as formulary items to detect treatment patterns, estimate the needs, ensure timely procurement, and prevent shortages/delays.
2. Move towards performance-based contracts, risk-sharing arrangements, or managed entry agreements with pharmaceutical companies that may be more favorable, as these take into consideration whether the drug is effective and safe.

#### Challenges in equitable access to cancer medications

Qatar has implemented a unique model that ensures equitable access to cancer medications. The government currently covers cancer treatment costs, including the cost of medications. However, once universal insurance coverage is introduced and mandated for all expatriate residents, it might generate new challenges in copayment rates and reimbursements, especially for patients with basic insurance packages, particularly for the costliest treatments.

Current cancer medications include targeted therapies, such as monoclonal antibodies and immune checkpoint inhibitors; however, patients need to be tested for genetic mutations to determine if they are eligible for such targeted therapies, which can sometimes be challenging due to the lack of some tests in Qatar and the need to send the samples abroad.

Other important challenges involve the availability of orphan medications for rare diseases, for example, in the field of hematology. Such medications may be urgently needed in critical situations. Although they are life-saving, sufficient quantities may not be kept in stock in Qatar and cannot be readily available within days if required.

Key recommendations:

1. Collaborate with pharmaceutical companies that provide drugs for targeted therapies to provide adequate tools and training for clinical and laboratory staff on biomarker testing and the reagents to be used.
2. Consider including the cost of testing in the overall treatment cost, specifically for targeted therapies.
3. Perform studies to identify the most common biomarkers present by type of cancer, specifically in the Qatari population. These may differ from biomarkers present in other populations and may help optimize patient-tailored approaches to guide therapeutic decisions.
4. Establish internal connections among Qatari institutions and establish external connections, networks, and hubs within the region, including hospitals throughout the Gulf Cooperation Council countries, that may facilitate and ensure the procurement of cancer drugs needed to address critical situations in a more expedited fashion.
5. Consider patients at the time of establishing insurance copayment rates to ensure that patients always have equitable access to cancer medications in accordance with the NCS.
6. Agree with QCS and other Qatari charities on special rates/discounts for cancer patients who cannot afford treatment.

#### Value-based options

Several value-based options are available in Qatar, and other models can also be adopted. The main issue with value-based practices is that value can mean different things depending on the interpreter. For instance, it can be interpreted as cost reduction, improved safety, clinical outcomes, patient adherence, quality of life, or patient satisfaction. Further, relying on traditional cost-effectiveness and cost-benefit research is very limiting as such research cannot assess values beyond clinical outcomes. Thus, it may not provide a complete picture of a drug’s comparative advantages or disadvantages [17].

Key recommendations:

1. Establish the definition of value concerning cancer treatment in Qatar.
2. Implement models that enable decision-making based on value according to current health economic principles, such as health technology assessment and multiple-criteria decision analysis, to enhance or complement the traditional cost-effectiveness studies commonly used to appraise cancer medicines in regional [15] and international settings [18].
3. Strengthen the local capacities and expertise needed to implement value-based decision-making models in terms of infrastructure, regulatory processes, and human resources.
4. Develop an implementation matrix that allows multiple-criteria decision analysis to select and include drugs as formulary items. The multiple-criteria decision analysis is a comprehensive, multi-dimensional approach that goes beyond cost-benefit and enables the evaluation of medications according to multiple criteria, including patient-reported outcomes, use for various indications, and frequency and ease of administration (e.g., taste/palatability for orally administered drugs), among other criteria, and is an analysis that has been successfully performed for other drug classes in Qatar [19, 20].
5. Raise awareness among healthcare providers about the need to consider value-based options.
6. Raise awareness about financial assistance and access programs for cancer patients, such as those offered by QCS.
7. Collect data about the financial toxicity of cancer treatments, available through QCS and other charities, to help understand the entire patient journey and find potential solutions.

### Treatment domain

#### Challenges in application/adoption of guidelines

Although Qatari oncology guidelines are typically aligned with international guidelines such as the American Society of Clinical Oncology (ASCO), European Society for Medical Oncology (ESMO), American Society of Hematology (ASH), European Hematology Association (EHA), and NCCN, the constantly evolving oncology practice and frequent emergence of new therapies approved by FDA/EMA as well as many guideline updates preclude these national guidelines from being updated promptly. Further, these guidelines cannot recommend non-formulary medications and drugs that are not readily accessible in the country.

Research has shown that international guidelines improve the quality of care [21–23]. The reduction in treatment costs, complications, and hospitalizations are attributable to the clinical pathways under the umbrella of the guidelines. HMC has developed local clinical guidelines and pathways available by cancer type and the type of management needed by multidisciplinary teams and approved by the Clinical Practice Guidelines committee. It is challenging to obtain all recommended molecular and genetic tests in a timely manner, which may preclude the early start of treatment, especially in the case of patients with targetable mutations for which there is a known effective treatment.

Currently, there is a lack of involvement of the patient voice and feedback in developing and implementing cancer management guidelines.

Key recommendations:

1. Continue periodically updating guidelines to reflect the most recently approved and recommended treatments abroad.
2. Increase clinician awareness of available clinical pathways.
3. Continue to monitor the implementation of clinical guidelines.
4. Ensure the availability of all biomarker and molecular tests required for prevalent cancers in Qatar, allowing precision medicine implementation and appropriate use of targeted therapies.
5. Maximize collaborations with pharmaceutical companies to facilitate genetic/molecular testing for targeted therapies, thereby allowing integration of genomic data with clinical decisions.
6. Involve patients and patient communities in developing and implementing cancer treatment guidelines, focusing on the concept of value-based care and including patient-centered/reported outcomes that are important to patients and reflect patient goals of treatment and aspirations related to their disease and life [24].

A barrier to using new treatments in Qatar in a timely fashion is that although some new treatments are already FDA/EMA-approved and recommended by reference guidelines, they are not readily available for use in Qatar because they have not yet gone through the processes of formulary inclusion, which can make the procurement process lengthy. Of note, non-formulary item usage is already in place, as discussed in the Regulatory Domain section.

Key recommendations:

1. Provide fast-track approval for drugs approved by the FDA/EMA and incorporated into reference treatment guidelines, especially in conditions with no alternative treatments.
2. Continue the use of needed drugs initially as non-formulary items, which allows the collection of real-world data, and insight into the local experience, with the subsequent submission of an application for addition to the hospital formularies.

#### Treatment burden from the patient perspective

Although there is no specific guidance for treating visitor patients, expatriate residents benefit from free cancer care and support for cancer treatment from several charitable organizations. Resident patients are not covered for treatment with non-formulary medications. While the approved formulary includes the vast majority of drugs needed for cancer treatment, it is impossible to include all drugs, especially those used for rare cancers. In such circumstances, patients are usually supported by charities such as QCS.

Key recommendations:

1. Create regulation that provides standard guidance for treating visiting cancer patients, including insurance coverage of oncology medications.
2. Involve patients and patient organizations such as QCS in the process of creating the above regulation.

### Patient domain

#### Awareness surrounding participation in clinical trials

Several methods can raise awareness among patients regarding involvement or participation in clinical trials. Providing patient education is critical, and it should be designed simply and straightforwardly to communicate the potential advantages and benefits of participation in a clinical trial via pamphlets or ads. Additionally, direct reporting from patients about their treatment outcomes in cancer clinical trials would be helpful to reduce the gap in knowledge regarding patient-reported outcomes in oncology care in Qatar. It has been shown that using these outcomes to inform clinical practice leads to clinically relevant improvements in patient outcomes [25].

#### Patient insights

The following key patient insights were highlighted. Further patient education is required to fully understand the medication prescribed and its potential side effects. Patient-reported outcomes need to be incorporated into the clinical pathways. A separate pediatrics registry should be developed as this population of patients has unique needs. Additionally, a pediatric cancer control plan should be implemented. Patients have noted concerns regarding the change to universal insurance coverage, and it is necessary to ensure equitable access to novel cancer drugs. It will be necessary to train cancer patients and survivors to be advocates and discuss with policymakers about coverage of oncology medications.

In the past, there was a general perception among patients that oncology care in Qatar was substandard. In fact, it was common for Qatari patients to seek medical attention in other countries. Currently, cancer care in Qatar meets the best international standards. More patients are staying and receiving their treatment locally, which has benefits such as reducing the stress of travel and allowing patients to have the comfort and assistance of family and friends while undergoing treatment. However, it is important to create awareness among medical teams regarding existing standard processes and the financial assistance available for non-formulary drugs for resident cancer patients and clarify the proper channels for requesting such assistance.

Key recommendations:

1. Consider patients’ input not only for evaluating care and value of care but also in pricing, as there is a willingness to negotiate pricing in a “pay by patient” approach. Further, lower pricing should be considered for patients receiving financial aid to cover their treatment by establishing collaborations between cancer hospitals, charities, and pharmaceutical companies.
2. Provide financial support to parents of pediatric patients who may have sacrificed their job to care for their child at home or in the hospital.
3. Conduct clinical research locally for a more personalized treatment that can be realistically delivered in Qatar without patients having to travel abroad to receive treatment.
4. Consider the value that the patient will gain from participating in a clinical trial in any awareness campaigns related to clinical trials.
5. Establish education programs or public education campaigns in collaboration with QCS to raise awareness for clinical trials and change the negative perceptions many patients have of participating in them.
6. Develop disease-specific support groups for patients through collaboration between HMC and QCS to improve adherence to cancer medications and promote the adoption and collection of patient-reported outcomes and participation in clinical trials.

## Study Limitations

There is lack of published data on similar work from the region except Saudi Arabia [15], where the healthcare system and population demographics, although have many similarities, are not exactly the same. We believe we had the best experts in Qatar (relevant to the study topic) in our panel, however expert consensus papers by nature are presenting the views of participating experts. Some other experts in the country may not agree with all our recommendations.

## Future Research

This paper is an eye-opening to the current practices on the access to novel cancer medications in Qatar from the experts’ point of view. Future work can be considered to address the patients’ viewpoints too as they are essential to have the full picture. In addition, a follow-up work to assess the implementation of this consensus paper recommendations would be very useful.

## Conclusion

This article summarizes the first consensus recommendations from the Q-OHEP meeting, focusing on four primary domains. These recommendations align with and build on those of the NCS and NCF and aim to provide a solid basis for evaluating, registering, approving, and reimbursing new cancer medications in order to accelerate patient access to novel cancer treatments in Qatar. In addition, they aim to promote and facilitate the adoption and collection of patient-reported outcomes as well as the implementation of value-based cancer care in Qatar. We hope to achieve the above goals using the structured recommendations reached by the consensus described herein.

## Data Availability

All data generated from the panel discussion and information analyzed during this study are included in this published article.

## Declarations

All authors gave electronic consent to participate in this study and have the virtual expert panel discussion recorded.

All methods were carried out in accordance with relevant guidelines and regulations.
